# Supplementary material for: Asynchronous digital health interventions for reviewing asthma: A mixed-methods systematic review protocol
Source: PLoS One. 2023 Feb 9;18(2):e0281538. doi: 10.1371/journal.pone.0281538 (PMC9910732; doi:10.1371/journal.pone.0281538)
Supplement: S1 Appendix — (DOCX) [file pone.0281538.s002.docx]

**S1 Appendix. Database search strategy**

**MEDLINE**

1 exp Asthma/

2 asthma$.ti,ab,kw.

3 exp Bronchial Spasm/

4 exp Bronchoconstriction/

5 exp Bronchial Hyperreactivity/

6 wheez$.ti,ab,kw.

7 1 or 2 or 3 or 4 or 5 or 6

8 exp Telemedicine/

9 exp Telenursing/

10 exp Electronic Mail/

11 exp Digital Technology/

12 exp Smartphone/

13 exp Mobile Applications/

14 (telehealth or teleconsult$ or telemanagement or telemonitor$ or telecare or telematics or telepharmacy or telehomecare or telesupport or ehealth or e-health or econsult$ or evisit$ or e-visit$ or mhealth or m-health or web or web-based or ict or digital questionnaire or portal).ti,ab,kw.

15 ((online or remote or distant or asynchronous) adj3 (review$ or monitor$ check-up or follow-up or care or consult$)).ti,ab,kw.

16 8 or 9 or 10 or 11 or 12 or 13 or 14 or 15

17 7 and 16 1674

18 limit 17 to yr="2001 -Current"

**Embase**

1 exp asthma/

2 asthma$.ti,ab,kw.

3 exp bronchospasm/

4 exp bronchoconstriction/

5 exp bronchus hyperreactivity/

6 wheez$.ti,ab,kw.

7 1 or 2 or 3 or 4 or 5 or 6

8 exp telemedicine/

9 exp telenursing/

10 exp teleconsultation/

11 exp e-mail/

12 exp digital technology/

13 exp smartphone/

14 exp mobile application/

15 (telehealth or telemanagement or telemonitor$ or telecare or telematics or telepharmacy or telehomecare or telesupport or ehealth or e-health or econsult$ or evisit$ or e-visit$ or mhealth or m-health or web or web-based or ict or digital questionnaire or portal).ti,ab,kw.

16 ((online or remote or distant or asynchronous) adj3 (review$ or monitor$ check-up or follow-up or care or consult$)).ti,ab,kw.

17 8 or 9 or 10 or 11 or 12 or 13 or 14 or 15 or 16

18 7 and 17

19 limit 18 to yr="2001 -Current"

**PsycInfo**

1 exp Asthma/

2 (asthma$ or bronchospasm or bronchoconstrict$ or "bronchial hyperreactivity" or wheez$).tw.

3 1 or 2

4 exp Telemedicine/

5 exp Teleconsultation/

6 exp Digital Technology/

7 exp Smartphones/

8 exp Mobile Applications/

9 (telehealth or telemanagement or telenurs$ or telemonitor$ or telecare or telematics or telepharmacy or telehomecare or telesupport or ehealth or e-health or econsult$ or evisit$ or e-visit$ or mhealth or m-health or web or web-based or email or e-mail or ict or digital questionnaire or portal).tw.

10 ((online or remote or distant or asynchronous) adj3 (review$ or monitor$ check-up or follow-up or care or consult$)).tw.

11 4 or 5 or 6 or 7 or 8 or 9 or 10

12 3 and 11

13 limit 12 to yr="2001 -Current"

**Scopus**

( TITLE-ABS-KEY ( asthma* OR bronchospasm OR bronchoconstrict* OR "bronchial hyperreactivity" OR wheez* ) AND TITLE-ABS-KEY ( telemedicine OR telehealth OR teleconsult* OR telemanagement OR telemonitor* OR telecare OR telematics OR telepharmacy OR telehomecare OR telesupport OR ehealth OR e-health OR econsult* OR e-consult* OR evisit OR e-visit OR mhealth OR m-health OR web OR "web-based" OR email OR e-mail OR ict OR "digital questionnaire*" OR portal  OR telenurs* OR "digital technology"  OR "smart phone" OR smartphone OR "mobile applications" OR ( ( online OR remote OR distant OR asynchronous ) W/3 ( review* OR monitor* OR check?up OR follow?up OR care OR consult* ) ) ) ) AND PUBYEAR > 2000 AND PUBYEAR > 2000

**CINAHL**

S1 TI asthma* OR bronchospasm OR bronchoconstrict* OR "bronchial hyperreactivity" OR wheez

S2 AB asthma* OR bronchospasm OR bronchoconstrict* OR "bronchial hyperreactivity" OR wheez*

S3 S1 OR S2

S4 TI telemedicine OR telehealth OR teleconsult* OR telemanagement OR telemonitor* OR telecare OR telematics OR telepharmacy OR telehomecare OR telesupport OR ehealth OR e-health OR econsult* OR e-consult* OR evisit OR e-visit OR mhealth OR m-health OR web OR "web-based" OR email OR e-mail OR ict OR "digital questionnaire*" OR portal OR telenurs* OR "digital technology" OR "smart phone" OR smartphone OR "mobile applications" OR ((online OR remote OR distant OR asynchronous) N3 (review* OR monitor* OR check?up OR follow?up OR care OR consult*))

S5 AB telemedicine OR telehealth OR teleconsult* OR telemanagement OR telemonitor* OR telecare OR telematics OR telepharmacy OR telehomecare OR telesupport OR ehealth OR e-health OR econsult* OR e-consult* OR evisit OR e-visit OR mhealth OR m-health OR web OR "web-based" OR email OR e-mail OR ict OR "digital questionnaire*" OR portal OR telenurs* OR "digital technology" OR "smart phone" OR smartphone OR "mobile applications" OR ((online OR remote OR distant OR asynchronous) N3 (review* OR monitor* OR check?up OR follow?up OR care OR consult*))

S6 S4 OR S5

S7 S3 AND S6

S8 S7 (Published Date: January 2001 – present)

**Cochrane library**

#1 (asthma* OR bronchospasm OR bronchoconstrict* OR "bronchial hyperreactivity" OR wheez*):ti,ab,kw

#2 (telemedicine OR telehealth OR teleconsult* OR telemanagement OR telemonitor* OR teleconsult* OR telecare OR telematics OR telepharmacy OR telehomecare OR telesupport OR ehealth OR e-health OR econsult* OR e-consult* OR evisit OR e-visit OR mhealth OR m-health OR web OR "web-based" OR email OR e-mail OR ict OR "digital questionnaire*" OR portal OR telenurs* OR "digital technology" OR "smart phone" OR smartphone OR "mobile applications" OR ((online OR remote OR distant OR asynchronous) NEAR/3 (review* OR monitor* OR check?up OR follow?up OR care OR consult*))):ti,ab,kw

#3 #1 AND #2

#4 #3 with Cochrane Library publication date Between Jan 2001 and present
